# Predicting the fatigue in Parkinson's disease using inertial sensor gait data and clinical characteristics

**DOI:** 10.3389/fneur.2023.1172320

**Published:** 2023-06-14

**Authors:** Hui Wang, Binbin Hu, Juan Huang, Lin Chen, Min Yuan, Xingfu Tian, Ting Shi, Jiahao Zhao, Wei Huang

**Affiliations:** ^1^Department of Neurology, The Second Affiliated Hospital of Nanchang University, Nanchang, China; ^2^Department of Neurology, Jiangxi Provincial People's Hospital, The First Affiliated Hospital of Nanchang Medical College, Nanchang, China

**Keywords:** Parkinson's disease, fatigue, gait, wearable inertial sensor, movement disorders

## Abstract

**Objectives:**

The study aimed to analyze the clinical features and gait characteristics of patients with Parkinson's disease (PD) who also suffer from fatigue and to develop a model that can help identify fatigue states in the early stages of PD.

**Methodology:**

A total of 81 PD patients have been enrolled for the Parkinson's Fatigue Scale (PFS-16) assessment and divided into two groups: patients with or without fatigue. Neuropsychological assessments of the two groups, including motor and non-motor symptoms, were collected. The patient's gait characteristics were collected using a wearable inertial sensor device.

**Results:**

PD patients who experienced fatigue had a more significant impairment of motor symptoms than those who did not, and the experience of fatigue became more pronounced as the disease progressed. Patients with fatigue had more significant mood disorders and sleep disturbances, which can lead to a poorer quality of life. PD patients with fatigue had shorter step lengths, lower velocity, and stride length and increased stride length variability. As for kinematic parameters, PD patients with fatigue had lower shank-forward swing max, trunk-max sagittal angular velocity, and lumbar-max coronal angular velocity than PD patients without fatigue. The binary logistic analysis found that Movement Disorder Society-Unified Parkinson's Disease Rating Scale-I (MDS-UPDRS-I) scores, Hamilton Depression Scale (HAMD) scores, and stride length variability independently predicted fatigue in PD patients. The area under the curve (AUC) of these selected factors in the receiver operating characteristic (ROC) analysis was 0.900. Moreover, HAMD might completely mediate the association between Hamilton Anxiety Scale (HAMA) scores and fatigue (indirect effect: β = 0.032, 95% confidence interval: 0.001–0.062), with a percentage of mediation of 55.46%.

**Conclusion:**

Combining clinical characteristics and gait cycle parameters, including MDS-UPDRS-I scores, HAMD scores, and stride length variability, can identify PD patients with a high fatigue risk.

## Introduction

Fatigue is typically defined as a persistent feeling of exhaustion that cannot be explained by the effects of drugs, medicine, or mental disorders, and it is a predictable and transient phenomenon that can be relieved by rest without interfering with daily activities (1). Pathological fatigue often exists during rest, which is a cause of falls and reduces the quality of life, and even worse, it significantly impairs the patient's mobility (2). Unfortunately, the perception of fatigue in patients with pathological fatigue may be chronic and unpredictable (3). Parkinson's disease (PD) is a degenerative disease of the central nervous system caused by the progressive loss of dopaminergic neurons and is classified as a movement disorder. PD symptoms can be classified into motor and non-motor. Its cardinal motor symptoms are tremor, rigidity, postural instability, and bradykinesia (4), while non-motor symptoms frequently include olfactory dysfunction, constipation, rapid-eye-movement sleep disorder, depression, excessive daytime sleepiness, cognitive impairment, psychiatric symptoms, autonomic nervous dysfunction, pain, and fatigue (5). James Parkinson noticed that patients had fatigue symptoms when he first proposed PD, and fatigue was first considered one of PD's non-motor symptoms in 1993 (6). Fatigue can exist in the early or pre-motor PD stages and becomes more pronounced as the disease progresses (7, 8). In 2016, Kluger et al. (9) proposed that the diagnostic criteria of PD with fatigue must have a significant decrease in energy levels or an increase in effort disproportionate to the level of attempted activity, and the symptoms must be present almost every day for nearly a month, or exist for most of a day. At present, the scales commonly used to evaluate fatigue associated with PD include the Fatigue Severity Scale (FSS) (10), the Multidimensional Fatigue Inventary-20 (11), the Modified Fatigue Impact Scale (12), and the Parkinson's Fatigue Scale (PFS-16) (13). A recent meta-analysis reported that the prevalence rate of PD with fatigue was nearly 50% (14). Such a high prevalence rate indicates that we need to pay attention to the phenomenon of fatigue symptoms in patients with PD, and it is necessary for us to analyze it deeply.

A previous study has shown that the gait characteristics of patients with mood disorders are specific (15). Growing evidence showed that, compared to PD with mild mood disorders, PD with severe mood disorders exhibited significantly lower step length and velocity but increased step length variability and step time variability (16). The basal ganglia and prefrontal cortex both took an important role in gait movement control and emotion (17). Liu et al. (18) indicated that the frontoparietal attention network played a crucial role in PD with fatigue by using arterial spin labeling perfusion functional magnetic resonance imaging. The latest study conducted on a cohort of PD with fatigue found that the supplementary motor area, which played a major role in motor planning and movement execution, was implicated (19). At the same time, it was mentioned that fatigue was positively correlated with depression, anxiety, and apathy in a study of fatigue and neurological triad symptoms (20). Therefore, we hypothesized that PD with fatigue also had unique gait characteristics. Nevertheless, previous studies mainly focused on the clinical features of PD with fatigue or gait impairment in PD with mood disorder. In our study, we first evaluated relevant clinical characteristics and gait parameters as indicative biomarkers.

**Figure 1:**
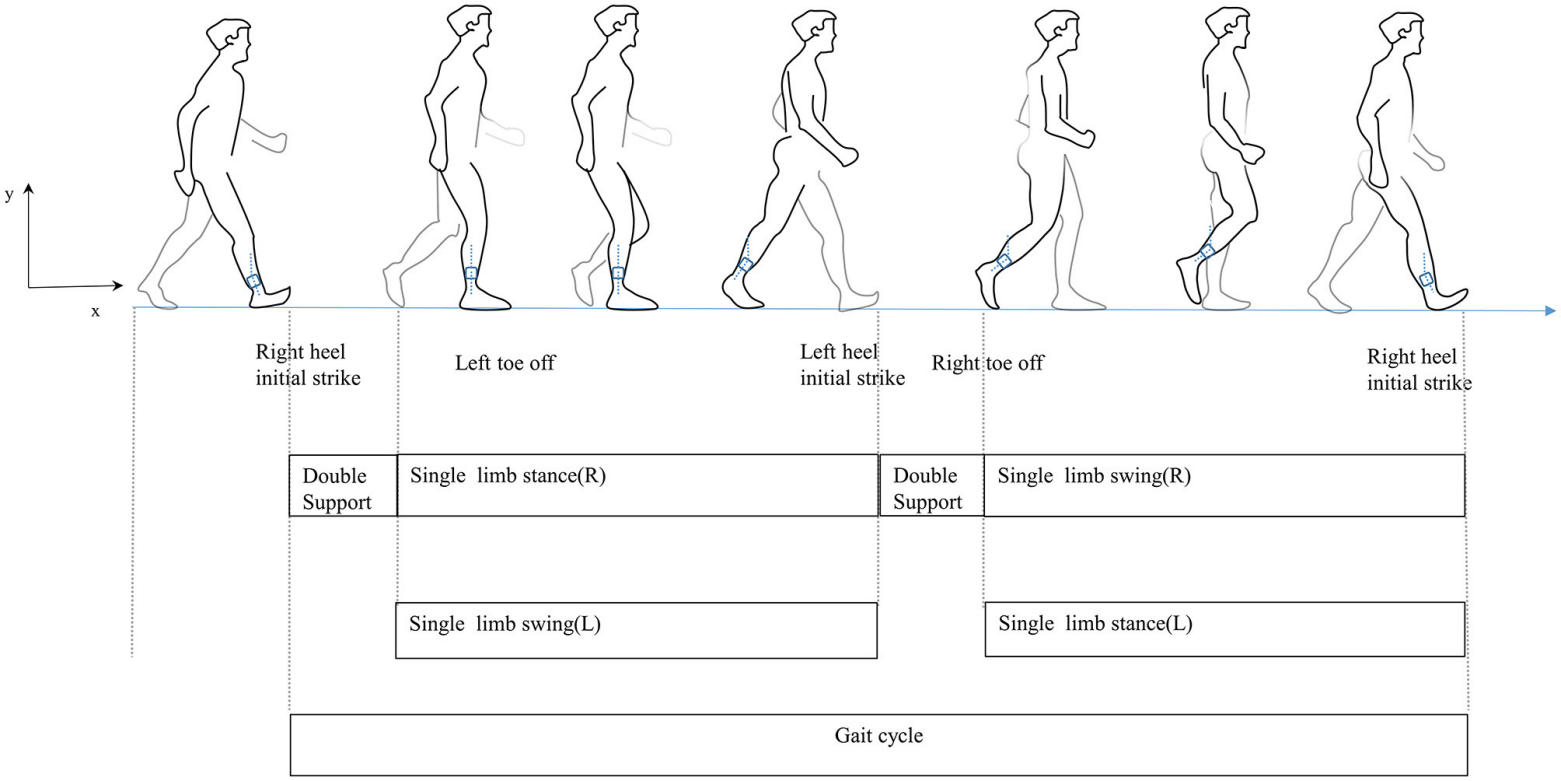
Human gait cycle.

Traditionally, wearable sensors used for gait analysis include inertial sensors (21), goniometers (22), and pressure and force sensors (23). Sensor-based wearable technology has advanced quickly recently. Their small size makes them easy to wear for long periods and facilitates gait data collection in everyday life. They provide technical support and have potential applications for exploring the relationship between PD and gait disorders mainly because they can objectively reflect the motor function and gait pattern of participants (24). The gait cycle is defined as the duration from initial contact to following contact on the same foot, including both the stance and swing phases (Figure 1) (25). In this study, the most easily performed action in daily life, the timed up and go, was used to characterize gait, as it has been widely used to acquire gait parameters in PD patients (26–28). Our study focuses not only on the clinical characteristics of PD with fatigue but also on the quantified gait parameters. We aim to analyze the relationship between clinical features and gait features and build a predictive model to draw clinicians' attention to PD with fatigue and conduct an early intervention.

## Materials and methods

### Subjects

Idiopathic PD patients who consecutively consulted the Second Affiliated Hospital of Nanchang University's PD-specialized outpatient clinic from August 2021 to December 2022 were collected as the PD group. All PD patients were evaluated during the mediation “off” period. The inclusion criteria for the PD group were: (a) patients with PD diagnosed according to the International Parkinson's and Movement Disorder Society (MDS) clinical diagnostic criteria; (b) both male and female patients with PD have to be older than 30 years; (c) Hoehn–Yahr stage ≤ 4; (d) MOCA score ≥15; (e) no skeletal muscle or muscle history of skeletal disease and no other causes affecting balance or gait (e.g., vertigo and fractures); and (f) stable dopaminergic medications for ≥1 month. The exclusion criteria were as follows: (a) patients with secondary Parkinson's disease; (b) patients with other neurological diseases; (c) patients with comorbid psychiatric diseases and other causes of fatigue (e.g., current or recurrent chronic disease, taking antidepressant drugs); and (d) patients who could not cooperate with clinical assessment. This study was approved by the Research Ethics Committee of the Second Affiliated Hospital of Nanchang University.

### Methodology

#### General data collection

During the consultation, demographic data (age, gender, height, and education), disease duration, medical history, basic physical examination, and the Unified Parkinson's Disease Rating Scale (MDS-UPDRS) were collected. Using Tomlinson et al. (29) algorithm, calculate the patient's daily levodopa equivalent dosage (LEDD). PD-related fatigue symptoms were assessed using the Parkinson's Fatigue Scale (PFS-16) recommended by the International Parkinson's and Movement Disorder Society (MDS) (9), a questionnaire consisting of 16 items in which subjects were asked to answer each item, ranging from 1 (strongly disagreed) to 5 (strongly agreed). Brown et al. (13) used a score of >3.3 to judge whether patients regarded fatigue as a problem, which was also the cutoff point for this study. According to the score of PFS-16, patients were divided into PD with (PD-F) and without fatigue (PD-NF). In addition, other non-motor indicators include daily living experience as assessed by MDS-UPDRS-I; cognitive function as assessed by the Montreal Cognitive Assessment (MoCA); anxiety as assessed by the Hamilton Anxiety Scale (HAMA); depression as assessed by the Hamilton Depression Scale (HAMD); daytime sleep behavior disorders as assessed by the Epworth Sleepiness Scale (ESS); and quality of life as assessed by the Parkinson's Disease Questionnaire (PDQ-39).

#### Analysis of gait parameters

**Figure 2:**
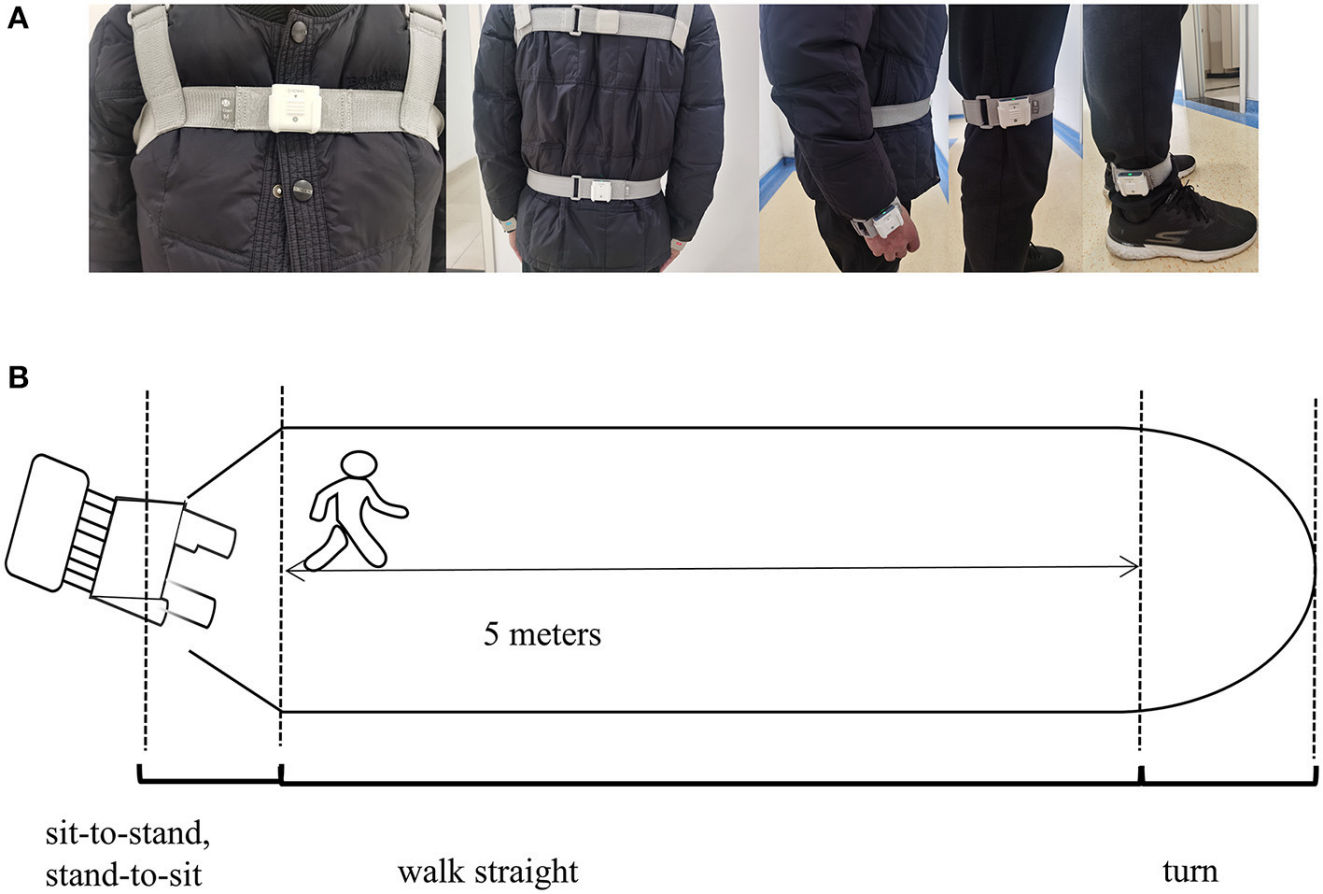
**(A)** View of the wearable device; **(B)** plan graph of the process of TUG.

The gait parameters were categorized into spatiotemporal and kinematic features. They were obtained using an inertial measurement unit (IMU) system (GYENOO Science, Shenzhen, China) at a sampling rate of 100 HZ (30). Ten IMU sensors were attached to the subject's lower back (L5), chest (sternum), bilateral wrists, thighs, ankles, and feet (Figure 2A). All subjects were asked to perform the Timed Up and Go Test (TUG): (1) sit quietly for 5 s; (2) get up from the chair; (3) walk straight for 5 m at a comfortable speed; (4) turn; (5) walk back, and (6) sit down quietly for 5 s (Figure 2B) (31). The start and end times of sitting and standing could be identified through the thigh pitch angle, and the change in waist horizontal rotation angle could identify the beginning and ending moments of the two turns. The data was then split into standing, straight walking, turning, and sitting segments. Only parts 3 and 5 were used in our study, and the number of gait cycles we used to estimate was about nine. Thirteen gait variables were estimated: step length, stride speed, cadence frequency, stride length, support phase, swing phase, shank front swing angle maximum, shank back swing angle maximum, shank angular velocity peak, trunk sagittal angular velocity peak, waist coronal angular velocity peak, step length variability, and stride length variability (Supplementary Figure 1, Supplementary Table S1 encompass the detailed definition). A coefficient of variation was applied to express variability: CV = $\frac{SD}{meanvalue}$.

### Statistical analysis

The data were statistically processed by SPSS 26.0 (IBM Corporation, USA). The Shapiro–Wilk test combined with Q-Q plots was used to determine the distribution of the collected data. We used the independent sample *t*-test for normally distributed continuous numerical variables and the non-parametric rank sum test for non-normally distributed continuous numerical variables. The chi-square test was used for categorical variables. Binary logistic regression analysis was used to determine which variables were good predictors of the presence of fatigue. Indices with significant differences in the independent sample *t*-test of clinical information and gait characteristics were selected as potential variables. The variance inflation factor (VIF) was used to assess multicollinearity, and variables had a VIF < 5. The regression equation was obtained by the backward stepwise regression method. Odds ratio (OR) values and 95% confidence interval (95% CI) were reported, and the model calibration was assessed by the Hosmer–Lemeshow goodness-of-fit. Moreover, the association between clinical features, gait parameters, and PDF-16 was studied using the Pearson correlation coefficient (r) (shown in Supplementary Table S2). A ROC curve was used to evaluate the sensitivity and specificity of the prediction model. We also used the SPSS macro program PROCESS (Model 4) (32) to test the mediating effects. A bootstrap estimation approach with 5,000 samples was used to measure the indirect effect. The mediating effect was considered significant when the 95% confidence interval (CI) did not contain zero. The complete mediating effect was considered when the total effect was significant, but the direct effect was not. In all data analyses, a *p*-value of < 0.05 was considered to be statistically significant. In order to reduce the type I error, the false discovery rate (FDR) method was used in this study, and we presented Δ*p* as *p*-values corrected by the FDR. The results with Δ*p* < 0.05 were regarded as statistically significant, while those with 0.05 < Δ*p* < 0.1 were considered a trend toward significance to increase the statistical power.

## Results

### Clinical baseline and clinical data of participants

According to the critical value recommended by the PFS-16 fatigue scale (score higher than 3.3), we divided PD patients into PD-F and PD-NF. The clinical and demographic data were shown in Table 1. In this study, 45.7% (37/81) of the patients were classified as suffering from fatigue. There were 23 males and 14 females in the PD-F group, with an average age of (65.11 ± 10.14) years. The PD-NF group included 25 males and 19 females, with an average age of (65.18 ± 7.99) years. There was no significant difference in gender, age, height, education level, and LEDD between the two groups (*p* > 0.05). Neuropsychological tests were used to collect clinical characteristics from patients. In the PD-F group, there were higher MDS-UPDRS-I scores (11.95 ± 5.70 vs. 7.11 ± 4.44, *p* < 0.001), higher MDS-UPDRS-II scores (14.46 ± 7.82 vs. 9.66 ± 6.38, *p* = 0.003), higher MDS-UPDRS-III scores (35.35 ± 16.97 vs. 26 ± 10.97, *p* = 0.040), higher HAMD scores (12 ± 7.59 vs. 6.5 ± 4.53, *p* < 0.001), higher HAMA scores (13.7 ± 8.76 vs. 9.32 ± 6.69, *p* = 0.013), higher ESS scores (10.16 ± 5.53 vs. 6.50 ± 3.74, *p* = 0.001), but lower PDQ-39 scores (37.32 ± 29.71 vs. 20.2 ± 19.41, *p* = 0.003).

**Table 1:** Demographic and clinical features of PD.

**Variables**	**PD-F (*n =* 37)**	**PD-NF (*n =* 44)**	**Statistic value**	***p* value**	**Δ*p* value**
Gender (female)	14 (37.83%)	19 (43.18%)	0.24	0.626^b^	0.743
Age, year	65.11 ± 10.14	65.18 ± 7.99	−0.04	0.971	0.971
Stature, m	1.63 ± 0.07	1.62 ± 0.07	0.32	0.751	0.793
Body mass, kg	58.81 ± 8.93	59.68 ± 10.81	−0.391	0.697	0.779
Education, year	7.38 ± 3.35	6.98 ± 3.76	0.50	0.617	0.740
Disease duration, year	6.0 (4,7.5)	4.53 (1.5,6)	542.50^a^	**0.010**	0.030
LEDD total, mg	558.85 ± 247.77	467.19 ± 287.76	1.52	0.132	0.217
L-Dopa	37 (100%)	41 (93.18%)	1.06	0.304^b^	0.525
DA	33 (89.19%)	44 (100%)	2.97	0.085^b^	0.179
MAO-B inhibitor	3 (8.11%)	1 (2.27%)	0.48	0.489^b^	0.715
MDS-UPDRS-part I, sores	11.95 ± 5.70	7.11 ± 4.44	4.29	**< 0.001**	< 0.001
MDS-UPDRS-part II, sores	14.46 ± 7.82	9.66 ± 6.38	3.04	**0.003**	0.014
MDS-UPDRS-part III, sores	35.35 ± 16.97	26.00 ± 10.97	2.99	**0.040**	0.095
MoCA, sores	21.14 ± 4.77	21.86 ± 5.35	−0.64	0.523	0.709
HAMD, sores	12.00 ± 7.59	6.50 ± 4.53	4.03	**< 0.001**	< 0.001
HAMA, sores	13.70 ± 8.76	9.32 ± 6.69	2.55	**0.013**	0.035
ESS, sores	10.16 ± 5.53	6.50 ± 3.74	3.43	**0.001**	0.006
PDQ39, sores	37.32 ± 29.71	20.20 ± 19.41	3.11	**0.003**	0.011

Data are expressed as mean ± SD or median (interquartile range) for continuous variables and percentage (%) for categorical variables as appropriate. ^a^Mann–Whitney U-test; ^b^χ^2^ statistic. Bold values indicate *p* < 0.05. MDS-UPDRS, Unified Parkinson's Disease Rating Scale; MoCA, Montreal Cognitive Assessment; HAMA, Hamilton Anxiety Scale; HAMD, Hamilton Depression Scale; ESS, Epworth Sleepiness Scale; PDQ-39, Parkinson's Disease Questionnaire; PD-F, Parkinson's disease with fatigue; PD-NF, Parkinson's disease without fatigue; LEDD, levodopa equivalent dose; DA, dopamine agonists; MAO-B, monoamine oxidase-B Δ*p, p-*value adjusted by false discovery rate.

### Spatiotemporal gait parameters

We collected spatiotemporal parameters such as step length, velocity, stride length, cadence, support phase, swing phase, and measured the variability of step length and stride length. The comparison of the spatiotemporal gait parameters between the two groups was shown in Table 2. We found that the PD-F group had significantly lower step length (0.42 ± 0.13 vs. 0.49 ± 0.12, *p* = 0.020), velocity (0.757 ± 0.27 vs. 0.867 ± 0.23, *p* = 0.049), and stride length (0.83 ± 0.27 vs. 0.97 ± 0.24), while the variability of step length (12.53 [6.12, 16.31] vs. 8.06 [4.56, 10.16], *p* = 0.029) and the variability of stride length (10.24 [4.69, 12.87] vs. 5.87 [3.43, 7.05], *p* = 0.015) were shown to be increased.

**Table 2:** Characteristics of spatiotemporal gait parameters in the PD with or without fatigue group.

**Variables**	**PD-F (*n =* 37)**	**PD-NF (*n =* 44)**	**Statistic value**	***p* value**	**Δ*p* value**
Cadence (steps/s)	1.84 ± 0.27	1.80 ± 0.17	0.913	0.364	0.485
Velocity (m/s)	0.757 ± 0.27	0.867 ± 0.23	−2.00	**0.049**	0.078
Step Length (m)	0.42 ± 0.13	0.49 ± 0.12	−2.37	**0.020**	0.053
Stride Length (m)	0.83 ± 0.27	0.97 ± 0.24	−2.43	**0.018**	0.072
Double Support (%)	22.71 ± 6.93	21.57 ± 5.5	0.82	0.413	0.413
Swing (%)	39.18 ± 3.53	39.68 ± 2.81	−0.72	0.472	0.472
CV-Step Length	12.53 [6.12, 16.31]	8.06 [4.56, 10.16]	583.00^a^	**0.029**	0.058
CV-Stride Length	10.24 [4.69, 12.87]	5.87 [3.43, 7.05]	437^a^	**0.015**	0.120

Data are expressed as mean ± SD or median (interquartile range) as appropriate; ^a^Mann–Whitney U-test; Bold values indicate *p* < 0.05; PD-F, Parkinson's disease with fatigue; PD-NF, Parkinson's disease without fatigue; Δ*p, p*-value adjusted by false discovery rate.

### Kinematic gait parameters

In our study, we acquired the kinematic parameters of shank-forward swing max, shank-backward swing max, shank-max sagittal angular, trunk-max sagittal angular velocity, lumbar-max coronal angular velocity, and we found that shank-forward swing max (14.70 ± 7.09 vs. 17.93 ± 6.80, *p* = 0.040), shank-max sagittal angular (274.93 ± 63.25 vs. 303.94 ± 56.17, *p* = 0.032), trunk-max sagittal angular velocity (27.21 ± 7.45 vs. 31.35 ± 8.62, *p* = 0.025), and lumbar-max coronal angular velocity (24.27 ± 9.38 vs. 31.87 ± 14.84, *p* = 0.009) were significantly different between the two groups (shown in Table 3).

**Table 3:** Characteristics of kinematic parameters in the Parkinson's disease with or without fatigue group.

**Variables**	**PD-F (*n =* 37)**	**PD-NF (*n =* 44)**	**Statistic value**	***p* value**	**Δ*p* value**
Shank-forward swing max (degree)	14.70 ± 7.09	17.93 ± 6.80	−2.08	**0.040**	0.050
Shank-backward swing max (degree)	−39.53 ± 7.09	−42.18 ± 5.97	1.74	0.086	0.086
Shank-max sagittal angular velocity (degree/sec)	274.93 ± 63.25	303.94 ± 56.17	−2.18	**0.032**	0.053
Trunk-max sagittal angular velocity (degree/sec)	27.21 ± 7.45	31.35 ± 8.62	−2.28	**0.025**	0.063
Lumbar-max coronal angular velocity (degree/sec)	24.27 ± 9.38	31.87 ± 14.84	−2.69	**0.009**	0.045

Data are expressed as mean ± SD or median (interquartile range) as appropriate; Bold values indicate *p* < 0.05; PD-F, Parkinson's disease with fatigue; PD-NF, Parkinson's disease without fatigue; Δ*p, p* value adjusted by false discovery rate.

### A predictive model of fatigue with PD

**Figure 3:**
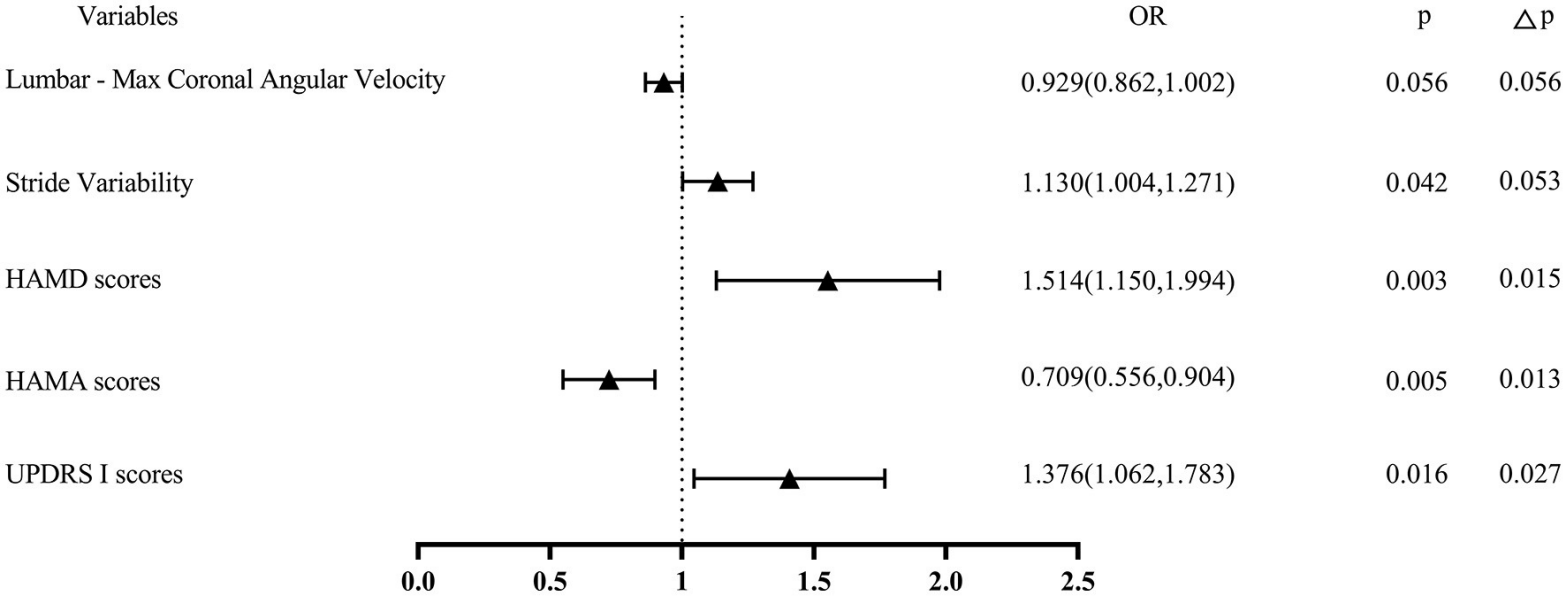
A binary logistic regression analysis of PD with and without fatigue. The odd ratio for each variable and 95% CI were reported and the p-values were shown on the right; Δ*p, p* value adjusted by false discovery rate.

**Figure 4:**
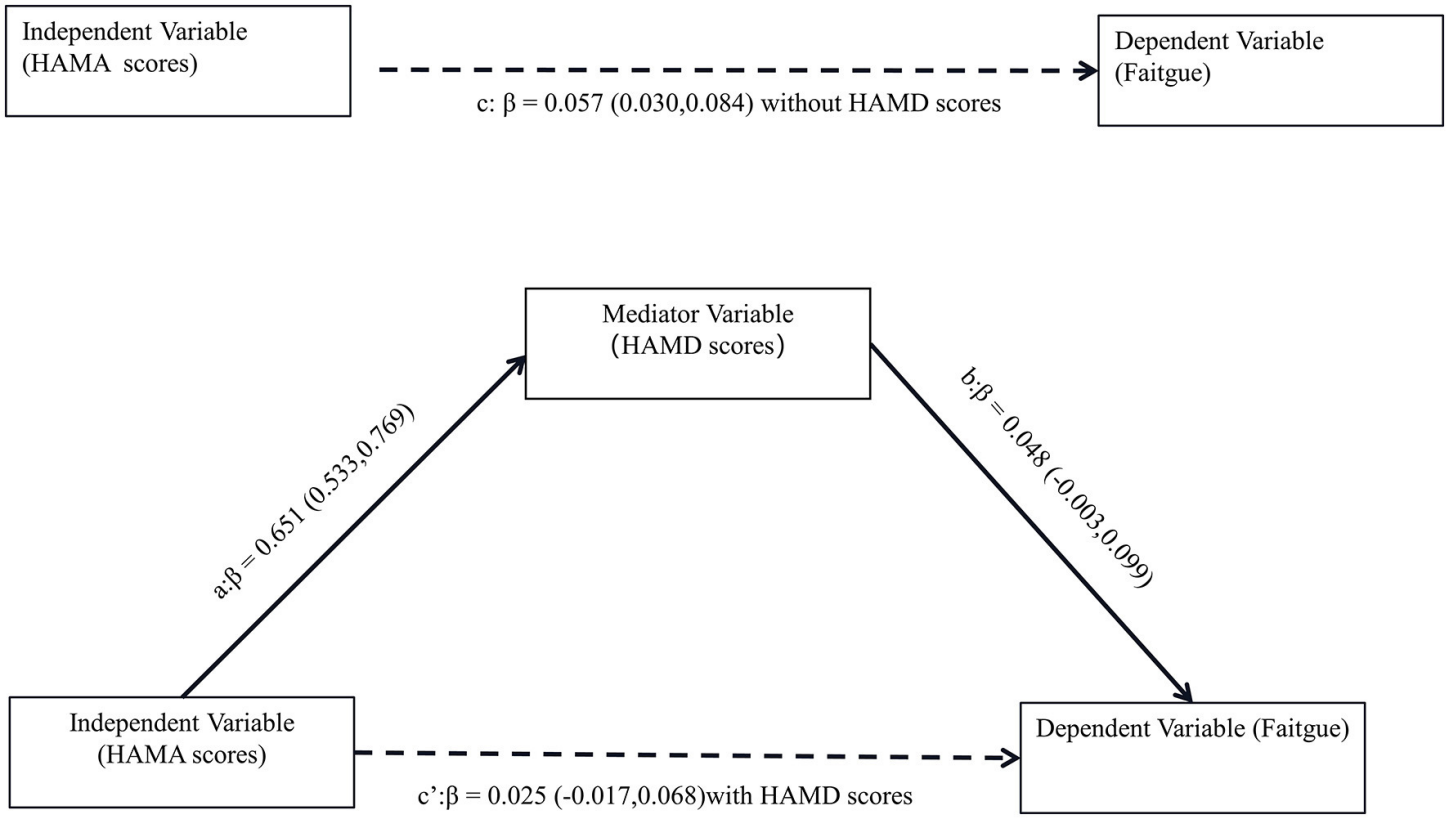
Mediation effect of depression on the association between anxiety and fatigue. Path c means the total effect, path c' means the direct effect and path a*b means indirect effect (β = 0.032,95% CI: 0.001,0.062), with a percentage of mediation of 55.46%.

**Figure 5:**
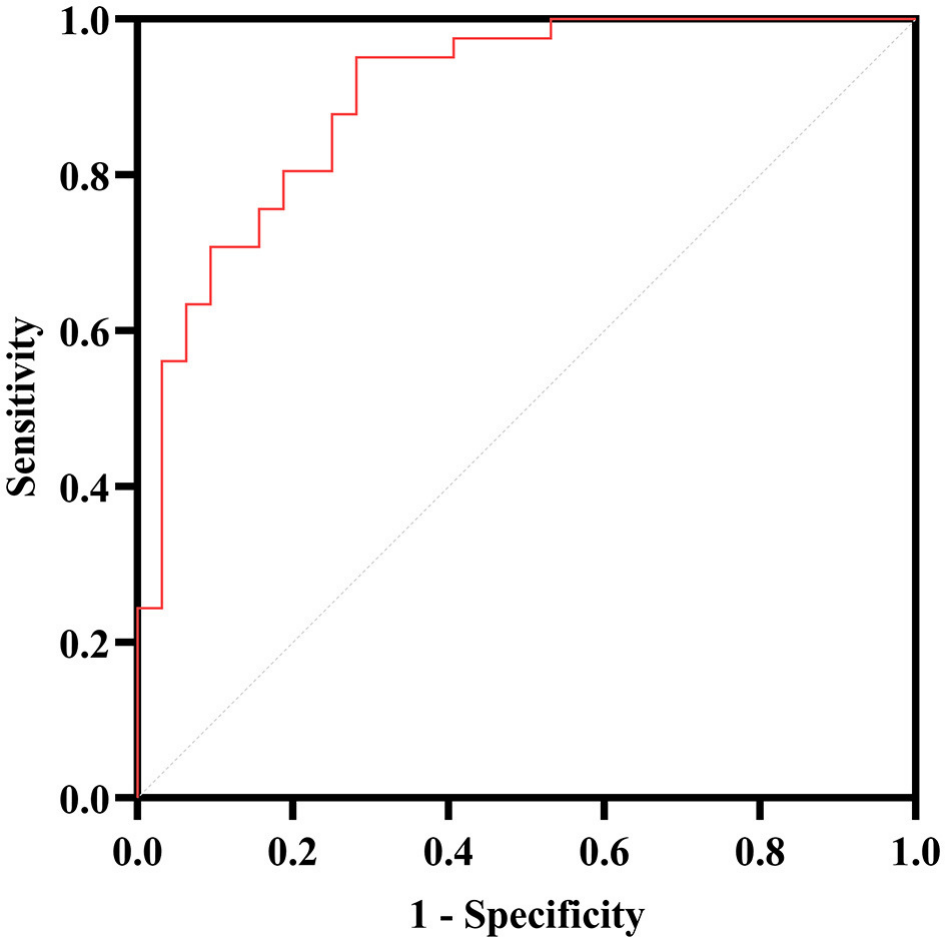
ROC curve analysis for the PD with fatigue. The AUC of the binary logistic predictive model (MDS-UPDRS-I, HAMA, HAMD, stride length variability and lumbar—max coronal angular velocity) is 0.900.

Afterward, the significantly different variables described above were identified as candidate variables. They were examined using binary logistic regression analysis, with OR values and 95% CI provided (Figure 3). MDS-UPDRS-I, HAMA scores, HAMD scores, and stride length variability were all found to be highly predictive of PD with fatigue (*p* < 0.05). Interestingly, an indirect path was found from HAMA (indirect effect = 0.032, *p* < 0.0001) to fatigue through HAMD. Moreover, as shown in Figure 4, HAMD may totally mediate the effect of HAMA on fatigue. The bootstrapped CIs of total and indirect effects were statistically significant, as shown in Table 4, with 55.46% of mediation. We also observed that the AUC was 0.900 (95% CI: 0.829–0.971), as shown in Figure 5. The following equation describes the probability (p) of developing PD with fatigue:

**Table 4:** Total, direct, and indirect effects of the mediation analysis investigating depression as a mediator between anxiety and fatigue.

				**Bootstrap 95%CI**		

	β	**Boot SE**	***p***	**Lower**	**Upper**	**P**_M_ **(%)**
Total effect	0.057	0.013	< 0.001	0.033	0.084	
Indirect effect	0.032	0.016	< 0.001	0.001	0.062	55.46%
Direct effect	0.025	0.021	0.242	−0.013	0.067	44.54%

CI, confidence interval; SE, standard error; P_M_, percentage of mediation.

Log (P/1-P) = −1.751 + 0.319 (MDS-UPDRS-I scores) – 0.344 (HAMA scores) + 0.415 (HAMD scores) + 0.122 (CV-Stride length) – 0.073 (Lumbar-Max Coronal Angular).

## Discussion

This study evaluated the PFS-16 of 81 patients and divided them into two groups based on the scores. The neuropsychological tests were used to assess the motor and non-motor symptoms of the two groups. The wearable device recorded the patients' gait characteristics.

### Differences in clinical symptoms between PD with and without fatigue

One of the most common non-motor symptoms of PD is fatigue. Our study observed that the PD-F group had the disease for longer than the PD-NF group. The MDS-UPDRS-I, which evaluated the psychological, behavioral, and emotional components, showed significant differences between the two groups (*p* < 0.001). Poltis and Niccolini (33) suggested that disrupting the serotonergic system, which regulates the sleep-awakening cycle, may contribute to daytime sleepiness and fatigue. In a one-year follow-up study of fatigue patients, Siciliano et al. (34) found that fatigue deterioration was delayed after treatment of daytime drowsiness and emotional apathy. Solla et al. (35) also found that mood/anxiety and daytime sleepiness were strongly correlated with fatigue. In this study, we inferred that the PD-F group had higher HAMA scores and that this was completely mediated by depression. Our findings showed that fatigued patients with PD have higher HAMA and HAMD scores than those without, suggesting that fatigue and mood are inextricably linked. Therefore, we further investigated the effects of the two factors on fatigue and found that depression was the mediating variable of the impact of anxiety on fatigue in the established mediation. Several studies have shown a negative relationship between fatigue and the ability to perform daily activities (36, 37). Consistent with previous research, the results of this study provided additional evidence. Using the MDS-UPDRS-II as an indicator for assessing daily activities and the PDQ-39 as an indicator for evaluating the quality of life, the PD-F group performed worse than the PD-NF group. Santos et al. (38) conducted a cross-sectional study on the relationship between non-motor and motor symptoms in patients with PD from the COPPADIS cohort. The results showed that patients with dyskinesia had higher total scores on the non-motor symptoms scale (NMSS), of which fatigue and sleep were the most significant. Consistent with previous research, this study also showed that the MDS-UPDRS-III score of the PD-F group (35.35 ± 16.97) was significantly higher than that of the PD-NF group (26 ± 10.97).

### Differences in gait performance between PD with and without fatigue

The gait of PD is characterized by a slowed gait speed (39), shortened step length (40), and impaired gait cadence (41). Mirelman et al. (42) noted that quantifying multiple gait characteristics under natural conditions can improve the sensitivity of gait quantification. Cook et al. (43) used TUG to assess motor function in patients with PD since its completion requires a static and dynamic balance. The pedunculopontine nucleus can regulate posture and gait control, as well as participate in the regulation of the sleep-awakening cycle, and it has been confirmed that the disturbance of the serotonergic system may cause fatigue (33, 44, 45). Thus, we hypothesized that patients with fatigue would have more significant differences in gait impairment than patients without fatigue in this study.

When subjects walk in a straight line at a comfortable pace, step length appears to be a good predictor of disease progression (46, 47). Furthermore, a 3-year follow-up study explored step length while performing dual tasks as an independent predictor of executive/attentive decline in patients (48). Although subjects in our study were not tested on cognitive tasks, it was also concluded that step length was significantly shorter in PD-F. Although significant in this study, gait speed was not specific to PD patients (47). The researchers emphasized the value of gait variability, especially in the early stages of the disease (49), and it has been proven to be a good predictor of fall risk in older people. A prospective study found that fatigue and falls were related to each other (50). Thus, we investigated the difference in step length variability and stride length variability between the two groups, and we found that they were both increased in PD-F.

Regarding kinematics, patients with PD were twice as likely to have an excessive trunk flexion posture as the general elderly population (51). Bertram et al. (52) also found that healthy elders compensate with trunk displacement in order to reach a distant glass of water, whereas PD patients did not adopt this compensatory strategy. In a sit-up trial, Wairagkar et al. (53) found that PD patients typically use a lower trunk acceleration to make postural alterations due to their standing instability and that fatigue exacerbates this performance (54). An earlier study suggested that trunk muscle strength was inversely related to angular velocity (55). The PD-F group required more muscle strength to meet the same standard task as the PD-NF group. Furthermore, our results showed that trunk-max sagittal angular velocity was lower in the PD-F group compared to the PD-NF group.

According to our study, PD with fatigue showed a lower lumbar-max coronal angular velocity. As coronal trunk stability during walking depended on active sensory integration and PD patients tended to show a disruption in the rhythmicity of pelvic acceleration, weakness seemed to aggravate this symptom (56, 57). This may explain why angular velocity was lower in PD patients with fatigue in our study.

The value of the oscillation of the shank in the forward-backward direction and the angular velocity reflect the distance the leg moved forward during the gait cycle (58). According to a study of 132 patients with PD, those with the postural instability and gait difficulty (PIGD) subtype showed more severe symptoms of fatigue (59). This discovery may partially explain why the PD-F group had a significantly lower shank-max sagittal angular than the PD-NF group.

### Potential prediction variables of PD with fatigue

Previous studies mainly focused on the relationship between clinical features and fatigue, and movement disorder specialists typically rely on patients' subjective feelings to identify fatigue and sometimes may ignore it. This study aimed to predict PD patients with fatigue by combining gait parameters obtained by an objective method with clinical characteristics. We found no difference between the two groups in terms of gender, age, height, education level, or LEDD at the baseline. We, therefore, took these variables out of the equation when predicting fatigue. In the aspects of clinical and gait parameters, we chose the lower *p-*value when the variables showed collinearity. A previous study revealed an association between fatigue and MDS-UPDRS (60). The MDS-UPDRS scores were taken into consideration as potential factors in our study and were calculated independently for parts I, II, and III. HAMA and HAMD scores were strong predictors of fatigue, as demonstrated by a prospective cohort study by Ou et al. (61). Gait variability was one of the descriptions of gait dysfunction (62), and a study conducted by Ibrahim et al. (63) showed that stride length and strike angle had a high correlation with fatigue. In our study, the predictive model showed that the HAMA scores, HAMD scores, MDS-UPDRS-I scores, and CV-stride variability were strong predictors of fatigue, the model fit using the Hosmer-Lemeshow goodness-of-fit test was 0.574 (*P* > 0.05), and the sensitivity and specificity of using the model to determine PD with fatigue were 71.9 and 95.1%, respectively.

## Limitation

This study has some limitations as well. First, the sample size included in this study was relatively small (*n* = 81), and the scale used for grouping is inherently subjective, which may have impacted the accuracy of the results. A larger sample size and further validation are necessary to establish reliability and objectivity. Second, crucial imaging indicators were left out of this study because it was carried out in an outpatient clinic and took patients' financial costs into account. Third, gait disorder in PD patients is more apparent when performing dual tasks. However, because this study aimed to improve the diagnosis rate of PD with fatigue by combining neurological scales and gait parameters, the most straightforward single task in daily life was adopted for the study. In future, we should still study gait parameters under different tasks or add imaging features, which may be of more diagnostic value. At the same time, it can also deepen our understanding of PD with fatigue. Future studies will focus on analyzing gait parameters in different task states. Fourth, we calculated the gait parameters from the beginning to the end of the test without considering the acceleration or deceleration phases. In future studies, it should be taken into consideration.

## Conclusion

In conclusion, the severity of motor symptoms, anxiety, and daytime sleepiness may exacerbate subjective fatigue and result in a poorer quality of life. Since fatigue is still identified using scales or subjective complaints from patients, a reliable method for identifying fatigue status in PD patients still needs to be improved. PD patients with fatigue have distinct gait characteristics. Therefore, we collected the parameters dynamically and continually using a new portable wearable device. We thoroughly examined the clinical characteristics of PD patients with fatigue. Then, we combined them with quantitative gait parameters to construct a prediction model that included MDS-UPDRS-I, HAMA, HAMD, and CV-Stride length. This is the first study to combine people's daily walking gait with clinical data to predict PD with fatigue. In future, it will help patients, clinicians, and caregivers improve their awareness of fatigue, so they can intervene as soon as possible and improve their quality of life.

## Data availability statement

The raw data supporting the conclusions of this article will be made available by the authors, without undue reservation.

## Ethics statement

The studies involving human participants were reviewed and approved by Research Ethics Committee of The Second Affiliated Hospital of Nanchang University. The patients/participants provided their written informed consent to participate in this study. Written informed consent was obtained from the individual(s) for the publication of any potentially identifiable images or data included in this article.

## Author contributions

HW and BH: analysis and interpretation of data and drafting and critical revision of the manuscript. JZ and WH: critical revision of this manuscript, supervision of this study, and obtained the funding. JH, LC, and MY: conduct data. XT and TS: enrollment of patients and collected the data. All authors contributed to the article and approved the submitted version.
