# Supplementary figures and images for: Predicting the fatigue in Parkinson's disease using inertial sensor gait data and clinical characteristics
Source: Front Neurol. 2023 Jun 14;14:1172320. doi: 10.3389/fneur.2023.1172320 (PMC10303817; doi:10.3389/fneur.2023.1172320)

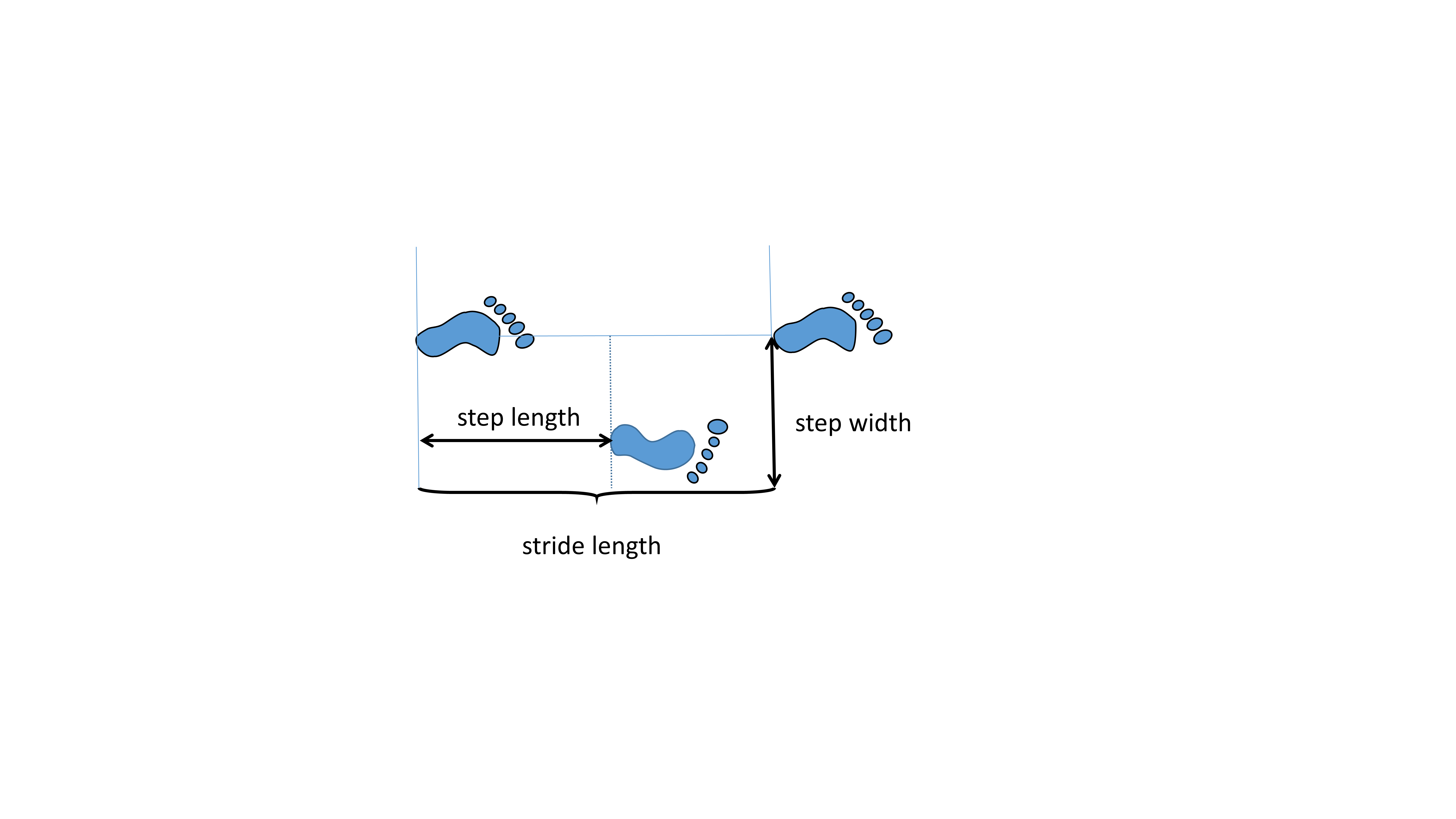

Supplement: Supplementary file 1 [file Image_1.TIF]
