# Supplementary material for: Predicting the fatigue in Parkinson's disease using inertial sensor gait data and clinical characteristics
Source: Front Neurol. 2023 Jun 14;14:1172320. doi: 10.3389/fneur.2023.1172320 (PMC10303817; doi:10.3389/fneur.2023.1172320)
Supplement: Supplementary file 2 [file Table_1.DOCX]

| Gait parameters | Definition | SI |
| --- | --- | --- |
| *Spatiotemporal gait parameters* | |  |
| Gait cycle | Interval between two consecutive strikes of the same heel during walking. |  |
| Step Length | Projection length of the straight distance between one foot following the ground and the other during walking on the sagittal plane of the human body. | m |
| Stride Length | The straight-line distance between two consecutive strikes of the same heel during walking. | m |
| Cadence | The number of steps taken in a unit of time | steps/s |
| Velocity | The ratio of all stride lengths to the time of the current gait cycle | m/s |
| Double Support | The sum of the time that both feet are in contact with the ground during one gait cycle. | % |
| Swing | The time the foot is in the air during one gait cycle. | % |
| *Kinematic parameters* | |  |
| Shank-Forward Swing Max | Maximum swing angle of shank forward in all straight stages. | degree |
| Shank-Backward Swing Max | Maximum swing angle of shank backward in all straight stages. | degree |
| Shank-Max Angular Velocity | Peak angular velocity of shank in all straight phases | degree/sec |
| Trunk-Max Sagittal Angular Velocity | Peak sagittal angular velocity of trunk in all straight phases. | degree/sec |
| Lumbar-Max Coronal Angular Velocity | Peak coronal angular velocity of lumbar in all straight phases. | degree/sec |

**Table S1 Gait parameters definition**

Note: Calculate the mean and standard deviation, respectively, based on each subject’s gait parameters acquired by the IMU system; the gait parameters were automatically calculated by the MATLAB algorithm. The details can be obtained from the website <https://www.gyenno.com/matrix-en> (Caveat: the validation reported on the website has not been peer reviewed and has only been tested on 13 healthy subjects); SI: International System of Units.
