# Supplementary material for: Predicting the fatigue in Parkinson's disease using inertial sensor gait data and clinical characteristics
Source: Front Neurol. 2023 Jun 14;14:1172320. doi: 10.3389/fneur.2023.1172320 (PMC10303817; doi:10.3389/fneur.2023.1172320)
Supplement: Supplementary file 3 [file Table_2.DOCX]

**Table S2 Pearson correlation (r) between the Parkinson Fatigue Scale and that of other measurements**

| Variables | Parkinson Fatigue Scale | | |
| --- | --- | --- | --- |
|  | r | P value | Δp value |
| *Clinical features* |  |  |  |
| Disease duration | 0.187 | 0.094 | 0.219 |
| MDS-UPDRS-part I, sores | 0.512 | < 0.001 | **< 0.001** |
| MDS-UPDRS-part II, sores | 0.403 | < 0.001 | **< 0.001** |
| MDS-UPDRS-part III, sores | 0.352 | 0.001 | **0.004** |
| HAMD, sores | 0.448 | < 0.001 | **< 0.001** |
| HAMA, sores | 0.423 | < 0.001 | **< 0.001** |
| ESS, sores | 0.454 | < 0.001 | **< 0.001** |
| PDQ39, sores | 0.345 | 0.002 | 0.006 |
| *Spatiotemporal Gait Parameters* |  |  |  |
| Cadence | 0.092 | 0.414 | 0.458 |
| velocity | -0.137 | 0.223 | 0.312 |
| Step Length | -0.17 | 0.130 | 0.210 |
| Stride Length | -0.171 | 0.128 | 0.224 |
| Double Support | 0.091 | 0.418 | 0.439 |
| Swing | -0.085 | 0.452 | 0.452 |
| CV-Step Length | 0.187 | 0.094 | 0.197 |
| CV-Stride Length | 0.192 | 0.103 | 0.197 |
| *Kinematic Gait Parameters* |  |  |  |
| Shank - Forward Swing Max | -0.117 | 0.296 | 0.366 |
| Shank - Backward Swing Max | 0.136 | 0.227 | 0.298 |
| Shank - Max Sagittal Angular Velocity | -0.154 | 0.170 | 0.255 |
| Trunk-Max Sagittal Angular Velocity | -0.193 | 0.084 | 0.221 |
| Lumbar-Max Coronal Angular Velocity | -0.097 | 0.387 | 0.452 |

Note: MDS-UPDRS: Unified Parkinson's Disease Rating Scale; HAMA: Hamilton Anxiety Scale; HAMD: Hamilton Depression Scale; ESS: Epworth Sleepiness Scale; PDQ-39: Parkinson's Disease Questionnaire;statistically significant results are in **bold**; Δp: p value adjusted by false discovery rate.
